# Hiding in Plain Sight: A Case of Intestinal Tuberculosis Masquerading as Benign Lower GI Bleeding

**DOI:** 10.1155/crgm/7499395

**Published:** 2026-07-23

**Authors:** Lavender Otom, Priyanka Panwar, Gerald Odong, Fatma Kassim, Patricia Okiro, Christopher Opio, Saleem Abdulkarim

**Affiliations:** ^1^ Department of Internal Medicine, Aga Khan University Hospital Nairobi, Nairobi, Kenya; ^2^ Department of Radiology, Aga Khan University Hospital Nairobi, Nairobi, Kenya; ^3^ Department of Pathology, Aga Khan University Hospital Nairobi, Nairobi, Kenya

**Keywords:** extrapulmonary TB, HIV, intestinal tuberculosis, lower gastrointestinal bleeding

## Abstract

**Background:**

Intestinal tuberculosis (TB) represents the most common form of abdominal TB, often affecting the ileocaecal region. Lower gastrointestinal bleeding is an uncommon presentation and may create significant diagnostic uncertainty, particularly in immunocompromised patients and in settings where inflammatory bowel disease is also encountered.

**Case Presentation:**

A woman in her sixties from Africa presented with recurrent diarrhoea and intermittent haematochezia over a 6‐month period. Initial endoscopic evaluation was nondiagnostic. Cross‐sectional imaging later demonstrated multifocal bowel involvement affecting both the small and large bowel, including ileocaecal and rectoanal disease, with associated abdominopelvic lymphadenopathy. Histopathology from repeat colonoscopy biopsy showed caseating granulomas and Ziehl–Neelsen‐positive acid‐fast bacilli, confirming intestinal TB. Subsequent HIV serology was positive, with a CD4 count of 55 cells/mm^3^ at diagnosis. The patient improved markedly following antituberculous therapy and subsequent antiretroviral treatment.

**Conclusion:**

This case highlights a diffuse presentation of intestinal TB without pulmonary involvement in a patient with newly diagnosed advanced HIV infection. It underscores the diagnostic challenge posed by overlap with more common causes of lower gastrointestinal bleeding and inflammatory bowel disease and the importance of integrating clinical, radiological, endoscopic and histopathological findings to achieve timely diagnosis and successful medical management.

## 1. Introduction

Tuberculosis (TB) is a preventable and largely treatable infectious disease caused by *Mycobacterium tuberculosis*, primarily affecting the lungs but capable of involving almost any organ system. The gastrointestinal tract represents one of the most frequent sites of extrapulmonary disease [1].

Since 2023, TB has reclaimed its position as the foremost cause of death from a single infectious agent, after being surpassed by coronavirus (COVID‐19) for the preceding three years [1]. The global TB burden in 2023 was estimated at 10.1 and 11.7 million cases, up from 10.4 million in 2021. In total, 8.2 million individuals were newly diagnosed in 2023, increasing from 7.5 million in 2022; 84% had pulmonary TB and 16% extrapulmonary TB (EPTB) [1, 2]. TB caused an estimated 1.25 million deaths, including 1.09 million among HIV‐negative individuals and 161,000 among those living with HIV. Kenya was among the high‐burden countries that collectively contributed to 87% of the global TB caseload in 2023 [1].

A 2016 Kenyan TB prevalence survey reported a national rate of 426 cases per 100,000 population, with higher disease burden among men, urban residents, those aged 25–34 years, and individuals over 65 [3]. By 2023, national TB cases rose to an estimated 124,000 cases, reflecting a 7.2% increase from the previous year. HIV/TB coinfection stood at 25%, with a concerning 19% mortality rate [4].

Abdominal TB, a subtype of EPTB, involves the gastrointestinal tract, peritoneum, abdominal solid organs, and lymphatic tissue [5, 6]. The gastrointestinal tract is the site most frequently involved in abdominal TB [5]. True incidence is difficult to determine, as concurrent pulmonary disease may mask abdominal involvement. In an HIV/TB multicentre cohort, EPTB accounted for 28% (756/2695) of all TB cases, with abdominal TB being the third most common site, affecting 11% (85/765) [7]. A retrospective observational study conducted in Pakistan reported abdominal TB in 21% of extrapulmonary cases, and 15%–25% of cases occur alongside pulmonary TB [6, 8, 9].

Risk factors for EPTB are generally similar to those observed in abdominal TB [8]. These include female gender, extremes of age (under 15 years or over 64 years), diabetes mellitus, solid organ transplants, antitumour necrosis factor therapy and HIV coinfection [5, 8–10]. HIV increases the risk roughly 30‐fold, particularly as CD4 counts decline [9]. Genetic polymorphisms in low molecular weight proteins 2 and 7 (LMP2 and LMP7), involved in MHC Class 1 antigen processing and CD8+ T‐cell activation, have been associated with increased susceptibility [10, 11]. Genome‐wide association studies (GWAS) may further clarify genetic contributions to disease pathogenesis.

Although intestinal TB is well recognised in TB‐endemic settings, its diagnosis remains difficult because its clinical, endoscopic and radiologic features frequently overlap with those of Crohn’s disease (CD), colorectal malignancy, and other causes of chronic diarrhoea or lower gastrointestinal bleeding. Bleeding is an uncommon manifestation and may initially be attributed to more benign anorectal pathology, thereby delaying diagnosis. This report describes a case of diffuse intestinal TB presenting with persistent diarrhoea and recurrent gastrointestinal bleeding in a patient with newly diagnosed advanced HIV infection, despite an initially nondiagnostic colonoscopy and no evidence of pulmonary TB. The case is clinically relevant because it illustrates how integrated clinical, radiologic, endoscopic and histopathologic assessment can help distinguish intestinal TB from competing diagnoses in settings where both TB and IBD are encountered.

## 2. Case Presentation

A woman in her sixties with an unremarkable medical history presented with haematochezia occurring on two distinct occasions, spaced 6 months apart. Her history included repeated bouts of loose stools, which were often followed by frank blood. She also reported nonspecific symptoms such as nausea (without vomiting), generalised abdominal cramping, epigastric pain and bloating. Over the past 6 months, she experienced a weight loss of 7 kg. She denied experiencing fever or night sweats, did not smoke or consume alcohol and reported no family history of malignancy. Additionally, she had never previously undergone treatment for TB.

On examination, she appeared unwell but was afebrile. She had pallor, though there was no jaundice or cyanosis. Abdominal examination revealed diffuse tenderness without any other significant findings, and the remainder of her systemic examination was unremarkable.

**FIGURE 1:**
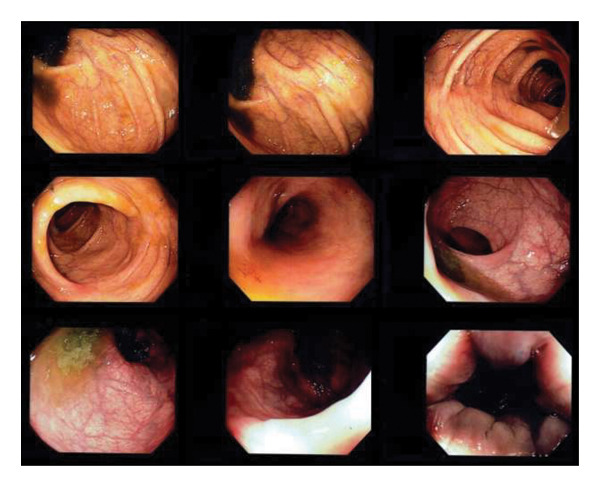
Initial colonoscopy only remarkable for Grade 2 haemorrhoids.

This was her second episode of lower gastrointestinal bleeding, prompting a review of her prior investigations. A colonoscopy performed during her first presentation revealed Grade 2 haemorrhoids but was otherwise unremarkable (Figure 1). A prior gastroscopy had shown corpus‐predominant gastritis. Given the worsening of her symptoms, including significant weight loss, she was admitted for further evaluation. Her haemoglobin was found to be 8.6 g/dL, with a reduced mean corpuscular volume (MCV) of 82.8 fL.

**FIGURE 2:**
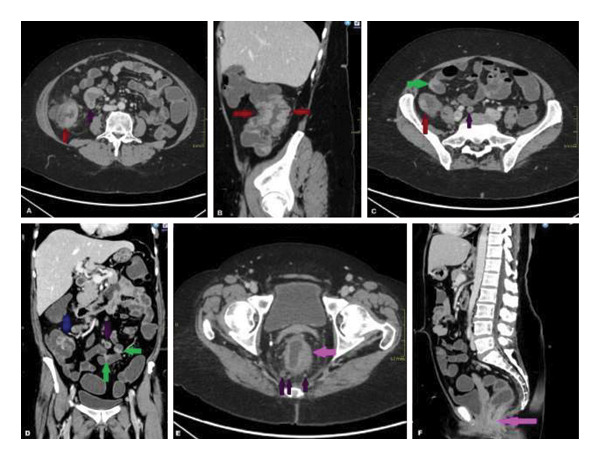
(A) Axial, post contrast CT showing eccentric noncircumferential enhancing mural thickening of the ascending colon (red arrow) along the mesenteric border with surrounding mesenteric fat stranding. Also shown is a conglomerate necrotic nodal mass (purple arrow) in the right iliac fossa is an ileocolic node; (B) sagittal contrast‐enhanced CT, again shown is the mural thickening and enhancement of the ascending colon (red arrows); (C) thickening of the ascending colon again shown (red arrow). Noncircumferential enhancing mucosal lesions of the distal ileum (green arrow) depicted in the right iliac fossa. Also shown is a necrotic right common iliac node (purple arrow); (D) coronal CT images showing mural thickening at the ileocecal valve (blue arrow). Multiple enhancing mucosal lesions in the mid ileum (green arrows) and nodes (purple arrow) are shown; (E, F) left eccentric hemi‐circumferential mural thickening of the low rectum (pink arrow) most prominent at 1 to 6 o’clock with mesorectal fat stranding and prominent mesorectal nodes.

**FIGURE 3:**
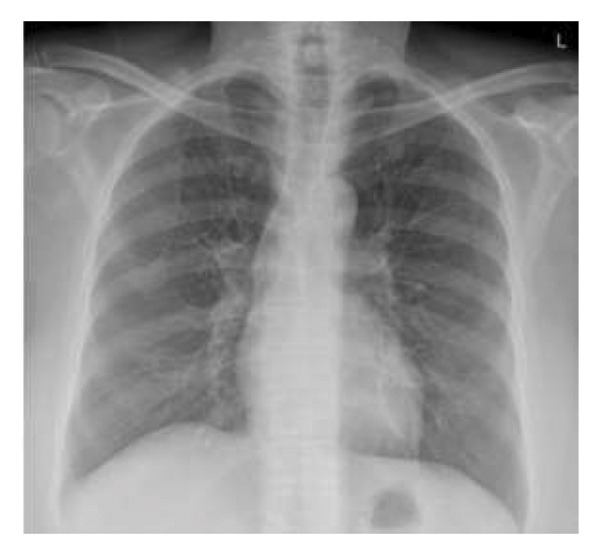
Normal chest radiograph.

Contrast‐enhanced abdominal CT revealed multifocal mucosal lesions in both the small and large bowel, affecting the mid and distal ileum as well as the ascending, transverse, descending, sigmoid colon and rectum (Figure 2). A dominant lesion centred at the caecum extended into the terminal ileum and proximal ascending colon, measuring up to 2 cm in maximal wall thickness and spanning approximately 6.6 cm in length. There was additional hemi‐circumferential thickening of the lower rectum extending through the anal canal, measuring up to 12 mm in thickness over a segment of approximately 6.3 cm. At the distal sites, there was evidence of transmural extension beyond the serosal surface into the surrounding perirectal fat, without involvement of the peritoneal reflections or mesorectal fascia. Innumerable abdominopelvic lymph nodes were noted, predominantly in pericolic regions corresponding to areas of bowel involvement, as well as within mesenteric and mesorectal stations. The largest was a conglomerate mesenteric nodal mass in the right iliac fossa measuring approximately 3.3 × 2.3 cm. Additional subcentimetre aortocaval and para‐aortic lymph nodes were also present, with the largest measuring 7 mm. Chest radiography showed no evidence of pulmonary TB (Figure 3).

**FIGURE 4:**
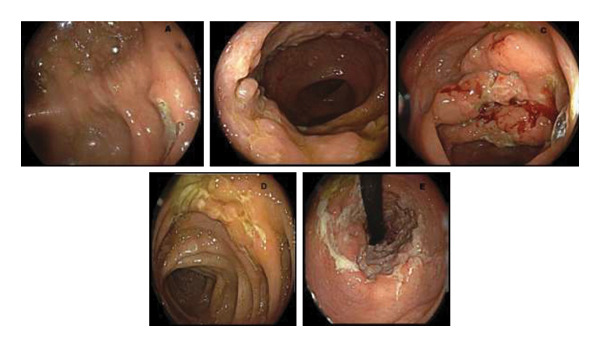
Colonoscopy images demonstrating irregular mucosal thickening and ulceration involving multiple colonic segments: proximal transverse colon (A), descending colon (B), sigmoid colon (C), and distal transverse colon (D). A circumferential ulceroproliferative lesion is noted in the rectum (E).

**FIGURE 5:**
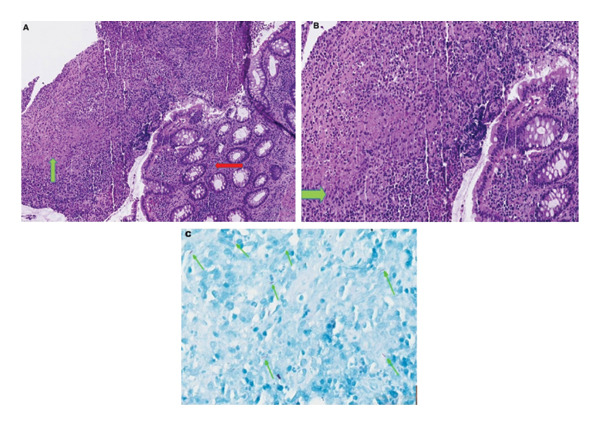
Colon biopsies: (A) low power magnification (X4) showing colonic mucosa (red arrow) with an ulcerated fragment within which is seen a necrotizing granuloma (green arrow); (B) medium power magnification (X10) showing the central area of necrotic debris composed of mixed acute on chronic inflammatory cells with peripheral epithelioid histiocytes; (C) high power magnification (X40) Ziehl–Neelsen stain showing acid‐fast bacilli (multiple green arrows).

A repeat colonoscopy showed multifocal transverse ulcers with irregular thickening involving the ascending, descending and transverse colon (Figure 4). There was extensive circumferential ulceration and thickening at the rectum just above the anal verge, accompanied by friable mucosa with overlying whitish exudate. Multiple biopsies were obtained, and the biopsy results confirmed the diagnosis of intestinal TB. Histological analysis revealed cryptitis, crypt abscesses and a marked infiltration of inflammatory cells within the lamina propria, including lymphocytes, plasma cells, neutrophils and eosinophils. No intranuclear nor cytoplasmic inclusions suggestive of CMV were noted, though CMV PCR was not performed on the biopsy specimen. Caseating granulomas were identified, and acid‐fast bacilli were demonstrated on Ziehl–Neelsen staining (Figure 5). TB culture and sensitivity, GeneXpert and drug susceptibility testing were not performed.

Subsequent HIV serology testing was positive with a CD4 count of 55 cells/mm^3^ at the time of diagnosis. Screening for other opportunistic infections, including a serum cryptococcal antigen test, toxoplasma immunoglobulin G levels, rapid plasma reagin and viral hepatitis markers, yielded negative results.

The patient was initiated on anti‐TB regimen (isoniazid, rifampicin, pyrazinamide and ethambutol), including pyridoxine supplementation, and 2 weeks later, highly active antiretroviral therapy (HAART) was initiated, consisting of tenofovir, dolutegravir (twice daily), lamivudine and cotrimoxazole. Throughout the intensive phase of treatment, the patient improved significantly clinically. Haematochezia and diarrhoea resolved, and her constitutional symptoms abated. She also regained weight. The patient was discharged under the care of the infectious disease clinic and has continued to do well on follow‐up. Follow‐up endoscopic and radiologic evaluations were not performed due to the patient’s preference.

## 3. Discussion

This case illustrates a diagnostically challenging presentation of intestinal TB as recurrent lower gastrointestinal bleeding in a patient with a newly diagnosed advanced HIV infection, without radiographic evidence of pulmonary TB. Although intestinal TB is well described, diffuse involvement of both the small and large bowel with rectoanal extension remains uncommon, and the initial attribution of bleeding to haemorrhoids contributed to delayed recognition. In addition, the combination of chronic diarrhoea, weight loss, multifocal bowel thickening, ulceration and immunosuppression created a broad differential diagnosis that included inflammatory bowel disease, colorectal malignancy and other infectious colitides. The case is therefore valuable not because it introduces a novel therapy but because it demonstrates how intestinal TB may remain occult despite early endoscopic assessment and how diagnosis can ultimately be secured through integration of clinical, radiologic, endoscopic and histopathologic findings.

Intestinal TB can arise from haematogenous or lymphatic spread, with the lungs serving as the primary site of *Mycobacterium tuberculosis* infection. Less frequently, transmission occurs via direct extension from nearby organs or through ingestion of contaminated food or sputum [5, 6, 12–14]. When *Mycobacterium tuberculosis* is consumed via contaminated milk or swallowed respiratory secretions, the bacilli penetrate the gastrointestinal tract through microfold (M) cells and dendritic cells, ultimately reaching the mucosal lining. There, they trigger the development of epithelioid granulomas within the submucosal lymphoid tissue [5, 6, 13]. Within 2 to 4 weeks, these granulomas undergo caseating necrosis, which results in ulceration of the overlying epithelial surface. The disease can then infiltrate the underlying layers and extend into neighbouring lymph nodes and the peritoneal cavity. In rare cases, the bacilli may enter the portal circulation or hepatic artery, leading to involvement of solid organs such as the liver, pancreas and spleen [6, 13]. In the absence of active pulmonary disease and without known risk factors such as the consumption of unpasteurised dairy products, haematogenous dissemination is the most probable source of infection in this scenario.

Gastrointestinal TB can be classified based on the site of involvement into the following categories: oesophageal, gastric, duodenal, jejunal, and ileocaecal, and colonic TB [6, 9, 13].

The ileocaecal region represents the most commonly affected site in gastrointestinal TB, responsible for roughly 64% of cases. This predisposition can be explained by several factors. First, the relatively prolonged faecal stasis in the ileocaecal region allows mycobacteria to have more time to adhere to and invade the mucosal surface [5, 6, 15, 16]. Additionally, this region contains a high density of lymphoid tissue, which plays a crucial role in immune response and is an ideal site for mycobacterial proliferation. Furthermore, the neutral pH environment in the ileocaecal area supports the survival and growth of *Mycobacterium tuberculosis* [6, 15, 16]. Lastly, the absorptive transport mechanisms in the gastrointestinal tract facilitate the uptake of swallowed mycobacteria, allowing them to pass through the mucosa and establish infection in the underlying tissues. These factors combined make the ileocaecal region particularly susceptible to TB infection [5, 6, 15, 16].

Isolated colonic involvement is observed in around 10.8% of gastrointestinal TB cases, with a higher frequency noted among individuals with compromised immune function. While the caecum is the predominant involved site, it is commonly affected as part of a continuous extension from the terminal ileum and ileocaecal junction. Multifocal colonic involvement, such as that reported here, is observed in 28%–44% of cases. Patterns of localised or segmental colonic involvement outside the caecum differ among studies. Some studies report the ascending colon as the most commonly involved segment, with subsequent involvement of the transverse colon and rectum, whereas other studies describe a sequential pattern beginning with the ascending colon, then involving the transverse and descending colon [6, 9, 17].

Intestinal TB occurs in three forms namely, the ulcerative variant (60%), the hypertrophic type (10%) and the ulcero‐hypertrophic pattern (30%) [12, 18, 19]. Clinical presentation is influenced by the host’s immune status. The ulcerative form is typically observed in individuals with immunosuppression, while the hypertrophic form is more common in those with a heightened immune response. In the ulcerative form, the characteristic feature is the presence of transverse ulcers, which are generally superficial and heal with fibrosis. However, deeper and circumferential ulcers may also develop, leading to stricture formation. In contrast, the hypertrophic variant is characterised by bowel wall thickening, fibrotic changes and scar formation, resulting in a firm, tumour‐like configuration that may resemble malignancy. The ulceroproliferative subtype, as observed in this case, combines both ulcerative and hypertrophic characteristics [12, 19, 20].

Lower gastrointestinal bleeding is reported in 5%–15% of individuals with intestinal TB [21–25]. It is primarily caused by extensive ulceration of the intestinal mucosa, which compromises the mucosal integrity and significantly increases the risk of haemorrhage [17, 18, 22, 24, 25]. The inflammation of the mucosa induced by the infection further exacerbates this risk [24]. Rarely, the formation of a mesenteric pseudoaneurysm due to TB vasculitis may underlie intestinal haemorrhage [17, 22, 25].

Clinical manifestations of intestinal TB often present with abdominal pain, fever, anorexia, unintended weight reduction, chronic diarrhoea, haematochezia and perianal disease [13, 23, 24, 26, 27]. The most frequent complication is intestinal obstruction, which can result from hyperplastic mural thickening, stricture formation or adhesions. Extraintestinal manifestations, such as arthralgia, aphthous ulcers, dermatologic and ocular abnormalities, may also be present. These symptoms closely resemble those of CD, making differentiation between the two conditions challenging [5, 6, 13, 23, 26, 28]. Although haematochezia is rarely observed in intestinal TB (occurring in 5%–20% of cases), it is more commonly seen in CD [18, 24, 26]. Abdominal complications associated with intestinal TB include intestinal obstruction, perforation, fistula formation, massive haemorrhage, enterolithiasis, traction diverticula and venous thrombosis [18, 22, 23, 25].

Although endoscopic findings in intestinal TB and CD frequently overlap, certain features such as transverse ulcers, a widened (patulous) ileocaecal valve and involvement of the caecum are more indicative of TB. Conversely, CD more often presents with disease in the left colon, longitudinal and aphthous ulcerations, as well as cobble‐stoning and skip lesions [6, 26, 28]. The presence of a dull white or yellow exudate covering ulcers, as observed in this case, has been documented, although it is not specific to intestinal TB [6, 13, 26]. Ulcers in intestinal TB are typically bordered by mucosa exhibiting signs of active inflammation, such as erythema, oedema, surface irregularities and nodular changes. In contrast, ulcers in CD are generally surrounded by mucosa that appears endoscopically normal [6, 26]. While both disorders involve chronic granulomatous inflammation, the granulomas found in intestinal TB are typically larger, confluent and dense with central caseation, a feature exclusive to TB [26]. Granulomas in CD are smaller, discrete and sparse. Submucosal granulomas with surrounding lymphocyte cuffing are more common in intestinal TB. Granulomas, regardless of caseation, are observed in less than 50% of patients, while aggregates of epithelioid cells without well‐formed granulomas are reported in 20%–30% of biopsies. A significant number of biopsies exhibit features of chronic inflammation but lack granulomas, caseation or clusters of epithelioid cells [26, 29].

The 6‐month interval between the patient’s first bleeding episode and definitive diagnosis is clinically important. Delayed diagnosis of abdominal TB has been reported in the literature and is often driven by the nonspecific nature of presenting symptoms, intermittent disease activity overlap with CD or malignancy and the limited diagnostic yield of initial endoscopy where lesions are patchy or evolving [13, 22–24, 26, 27]. In this patient, the initial colonoscopy demonstrated only haemorrhoids, a finding plausibly anchored clinical reasoning toward a benign anorectal source of bleeding. The subsequent emergence of persistent symptoms, weight loss, anaemia and cross‐sectional imaging abnormalities prompted re‐evaluation and repeat biopsy, which proved decisive. This sequence highlights the need for clinicians in TB‐endemic settings to reconsider intestinal TB when apparently benign lower gastrointestinal bleeding is recurrent or accompanied by constitutional symptoms, chronic diarrhoea, anaemia or multifocal bowel abnormalities.

A range of alternative conditions should be included in the differential diagnosis when evaluating intestinal TB based on endoscopic appearance. These include infectious diseases such as amoebiasis, *Yersinia* infection, gastrointestinal *Histoplasmosis* and peri‐appendiceal abscesses, as well as noninfectious conditions like intestinal Behçet’s disease and neoplasms [13, 17, 20, 26, 28, 29].

Microbiological tests, encompassing acid fast bacillus (AFB) smear, culture, polymerase chain reaction (PCR) and Gene Xpert MTB/Rif, can aid in the diagnosis, although their sensitivity remains relatively low. AFB smear positivity ranges from 2.7% to 37.5%, while culture can range from 2.7% to 70%. PCR for AFB has 44% sensitivity and 95% specificity, while Gene Xpert MTB/Rif has 8.1% sensitivity and 100% specificity [26].

In the present case, diagnosis relied primarily on histopathology and Ziehl–Neelsen staining of repeat colonoscopic biopsies, which demonstrated caseating granulomas and acid‐fast bacilli. Although additional microbiologic testing such as mycobacterial culture, GeneXpert, or PCR may strengthen diagnostic confirmation and provide drug susceptibility information, these were not performed in this case. This represents a limitation. However, the tissue findings, compatible endoscopic appearance, characteristic imaging abnormalities, absence of an alternative unifying diagnosis, and subsequent clinical response to antituberculous therapy together strongly supported the diagnosis of intestinal TB.

Imaging is adjunctive in the diagnosis of abdominal TB. Abdominal ultrasound can reveal concentric thickening of the ileocaecal region, which, when pronounced, creates a pseudo‐kidney appearance. Barium studies now have limited utility due to both their lower diagnostic accuracy and high radiation dose and have largely been superseded by cross‐sectional CT enterography. This imaging modality is capable of visualising the luminal surface, bowel wall, and extraluminal features of intestinal TB. MR enterography serves as a valuable adjunct, particularly for patients requiring multiple scans, as it reduces the cumulative radiation exposure. Active ileocaecal TB on CT or MR enterography is characterised by concentric, symmetrical mural thickening affecting the terminal ileum, ileocaecal valve, or caecum [13, 17, 26]. These imaging techniques are preferred for distinguishing CD from intestinal TB, as they overcome some of the limitations of endoscopy, particularly restricted access to areas of the small intestine beyond the distal ileum or proximal jejunum, and challenges in assessing regions located on either side of a stricture [13, 26].

CT abdomen is instrumental in evaluating luminal and extraluminal disease, including peritoneal and mesenteric disease, lymph node enlargement, and visceral organs. It also plays a key role in identifying complications of TB, such as abscesses, perforation, strictures, and bowel obstruction. Additionally, CT abdomen is valuable in diagnosing conditions that may mimic abdominal TB [17, 28]. Notably, about 38% of individuals diagnosed with abdominal TB also present with active pulmonary or miliary TB. Therefore, obtaining a CT thorax in the same imaging session can be particularly helpful [13, 17].

Surgical intervention plays a limited role in the diagnostic process for intestinal TB. However, in the presence of extraluminal disease, either laparoscopy or laparotomy can be performed to obtain biopsy specimens or fluid for further evaluation [13, 28]. Excisional biopsies can serve both diagnostic and therapeutic purposes, particularly in cases of complications like strictures, abscesses and perforation.

Treatment guidelines for intestinal TB recommend initiating antitubercular therapy. This typically involves an intensive 2‐month phase with isoniazid, rifampicin, pyrazinamide, and ethambutol, followed by a continuation phase of 4 months of two‐drug therapy with isoniazid and rifampicin. At baseline, it is beneficial to evaluate potential resistance to isoniazid and rifampicin, as treatment failure may occur in cases of drug resistance. A randomised controlled trial by Sang Hyoung et al. addressed whether treatment should extend beyond 6 months. The study found no difference in outcomes between groups treated for 6 months versus those treated for 9 months, suggesting that shorter treatment durations are favoured due to improved adherence and reduced risk of medication‐related adverse effects [5, 13, 30]. While there are no clear guidelines for follow‐up, patients on treatment should be monitored for potential medication toxicities and to evaluate treatment effectiveness.

In cases where patients are started on empiric antitubercular therapy guided by clinical presentation, imaging studies and histological findings that are suggestive but not definitive for TB, repeat endoscopy is recommended after 8–12 weeks to evaluate mucosal response to antitubercular therapy [13, 17, 26]. If there is no improvement, other conditions mimicking TB should be considered, and appropriate management should follow. When there is a clinical suspicion of TB, patients should not be started on steroids or other immunosuppressive drugs due to the risk of disease exacerbation, which could be life‐threatening [24, 26, 28].

Among individuals living with HIV, the timing of ART initiation is guided by the CD4 count and clinical condition. For those with a CD4 count < 50 cells/mm^3^, ART should be started within two weeks of beginning antituberculosis therapy (ATT). For patients with a CD4 count ≥ 50 cells/mm^3^, ART can typically be initiated within 8–12 weeks of ATT. However, if a patient with a CD4 count ≥ 50 cells/mm^3^ presents with severe disease, evidenced by factors such as a low Karnofsky performance score, reduced BMI, anaemia, hypoalbuminemia, organ dysfunction or extensive disease, ART should be initiated within 2–4 weeks of ATT initiation [31, 32]. In this case, ART was commenced within 2 weeks of ATT initiation. This strategy aims to reduce the likelihood of TB‐associated immune reconstitution inflammatory syndrome (TB‐IRIS) while also ensuring early detection and treatment of other opportunistic infections, which are more common in severely immunocompromised individuals and can further increase the risk of IRIS.

Surgery in intestinal TB is primarily reserved for managing complications like bowel obstruction, strictures or perforations [28]. When patients present with lower gastrointestinal bleeding, colonoscopy should be performed when feasible, with efforts made to control the bleeding through endoscopic means. If bleeding is massive and expertise is available, angiography and embolisation of the involved vessel may be considered. In some situations, surgical intervention may be necessary as a last resort [22, 24, 25, 28].

From a practical standpoint, this case supports a low threshold for repeat evaluation when symptoms persist despite an initially nondiagnostic endoscopy, particularly in TB‐endemic settings and in patients with suspected or confirmed immunosuppression. Cross‐sectional imaging can help identify multifocal bowel disease and nodal involvement beyond the reach of limited mucosal inspection, whose repeat targeted biopsy may increase diagnostic yield. Early HIV testing is also essential in patients with unexplained gastrointestinal disease suggestive of infection, as identification of advanced immunosuppression may substantially alter the differential diagnosis, urgency of investigation and treatment planning.

## 4. Limitations

A limitation of this case is that biopsy specimens obtained at repeat colonoscopy were submitted for histopathological assessment only and were not additionally sent for microbiological testing, such as TB culture, GeneXpert or CMV PCR. In retrospect, submitting tissue for both histology and microbiology would have strengthened the diagnostic work‐up and represents an important learning point from this case.

## 5. Conclusion

Diagnosing intestinal TB remains challenging because its presentation may be nonspecific and can closely mimic benign anorectal disease, inflammatory bowel disease, malignancy and other infectious colitides. In this patient, recurrent haematochezia was initially attributed to haemorrhoids, contributing to a 6‐month diagnostic delay despite appropriate early evaluation. This case highlights an uncommon diffuse presentation of intestinal TB involving both the small and large bowel, including rectal disease, in the absence of pulmonary TB in a patient with newly diagnosed advanced HIV infection. It underscores the importance of maintaining a high index of suspicion for intestinal TB in TB‐endemic settings when lower gastrointestinal bleeding is recurrent or accompanied by chronic diarrhoea, weight loss, anaemia, or multifocal bowel abnormalities. Integration of clinical, radiologic, endoscopic, and histopathologic findings was critical in establishing the diagnosis and enabled successful medical management without the need for surgery or endoscopic re‐intervention.

## Author Contributions

Lavender Otom and Priyanka Panwar contributed to the drafting and writing of the manuscript. Gerald Odong was responsible for the radiological interpretation and reporting of imaging findings. Fatma Kassim and Patricia Okiro contributed to the histopathological analysis and reporting. Christopher Opio supervised the preparation of the manuscript. Saleem Abdulkarim was responsible for patient management and also supervised the manuscript.

## Funding

No funding was received for this manuscript.

## Disclosure

All authors reviewed and approved the final version of the manuscript.

## Ethics Statement

The Institutional Review Board approved publication of this case report and waived the requirement for informed consent because the patient could not be contacted despite documented attempts. All potentially identifying information has been removed to minimize risk of identification.

## Conflicts of Interest

The authors declare no conflicts of interest.

## Data Availability

All data supporting the findings of this case report, including clinical details and images, are fully included within the published article. No additional data are available.
